# Self-injected contraceptives: does the investment reflect women’s preferences?

**DOI:** 10.1136/bmjgh-2022-008862

**Published:** 2022-07-14

**Authors:** Shannon N. Wood, Sophia Magalona, Linnea A. Zimmerman, Funmilola OlaOlorun, Elizabeth Omoluabi, Pierre Akilimali, Georges Guiella, Peter Gichangi, Philip Anglewicz

**Affiliations:** 1 Department of Population, Family and Reproductive Health, Johns Hopkins Bloomberg School of Public Health, Baltimore, Maryland, USA; 2 Department of Community Medicine, College of Medicine, University of Ibadan, Ibadan, Nigeria; 3 Department of Statistics and Population Studies, University of the Western Cape, Bellville, South Africa; 4 Kinshasa School of Public Health, Kinshasa, Congo (the Democratic Republic of the); 5 Institut Supérieur des Sciences de la Population/University of Ouagadougou, Ouagadougou, Burkina Faso; 6 International Centre for Reproductive Health – Kenya (ICRHK), Mombasa, Kenya

**Keywords:** Public Health, Cross-sectional survey, Health systems

## Abstract

Subcutaneous depot medroxyprogesterone acetate (DMPA-SC) is an innovative contraceptive method aimed at meeting women’s unique circumstances and needs, largely due to its ability to be self-injected. Substantial research and advocacy investments have been made to promote roll-out of DMPA-SC across sub-Saharan Africa. To date, research on the demand for DMPA-SC as a self-injectable method has been conducted largely with healthcare providers, via qualitative research, or with highly specific subsamples that are not population based. Using three recent rounds of data from Performance Monitoring for Action, we examined population-representative trends in demand, use, and preference for self-injection among current non-users in Burkina Faso, the Democratic Republic of Congo (Kinshasa and Kongo Central regions), Kenya, and Nigeria (Lagos and Kano States). We found that while over 80.0% of women had heard of injectables across settings, few women had heard of self-injection (ranging from 13.0% in Kenya to 24.8% in Burkina Faso). Despite initial increases in DMPA-SC prevalence, DMPA-SC usage began to stagnate or even decrease in all settings in the recent three years (except in Nigeria-Kano). Few (0.0%–16.7%) current DMPA-SC users were self-injecting, and the majority instead were relying on a healthcare provider for administration of DMPA-SC. Among current contraceptive non-users wishing to use an injectable in the future, only 1.5%–11.4% preferred to self-inject. Our results show that self-injection is uncommon, and demand for self-injection is very limited across six settings, calling for further qualitative and quantitative research on women’s views on DMPA-SC and self-injection and, ultimately, their contraceptive preferences and needs.

**Summary box**

- While most women had heard of injectables across all settings (ranging from 81.1% in Democratic Republic of the Congo (DRC)-Kongo Central to 92.4% in Kenya), far fewer women knew of the option to self-inject (ranging from 13.0% in Kenya to 24.8% in Burkina Faso).
- In all settings except Nigeria-Kano, subcutaneous depot medroxyprogesterone acetate (DMPA-SC) use had stagnated or declined over time.
- For the majority of users, DMPA-SC was administered by a professional healthcare provider; small proportions of women reported self-injection (0.0%–16.7%).
- Only 1.5%–11.4% of current non-users of contraception who intended to use the injectable in the future stated that they would prefer to self-administer the injection.

## Introduction

Innovations in contraceptive technology and delivery are needed to meet the unique needs and circumstances of women, men, and couples. One recent contraceptive innovation offers promise: subcutaneous depot medroxyprogesterone acetate (DMPA-SC), which is often known by its brand name, Sayana Press.^1^ Since 2014–2015, DMPA-SC has been gradually rolled out across sub-Saharan Africa; as of April 2022, approximately 50 countries offer DMPA-SC, and approximately 30 countries are currently piloting or scaling up self-injection of DMPA-SC.^2^

DMPA-SC is positioned to become a leading contraceptive method in sub-Saharan Africa because it offers several advantages over other contraceptive methods, particularly intramuscular depot medroxyprogesterone acetate (DMPA-IM).^3^ One particularly noteworthy attribute of DMPA-SC is that it comes in an easy-to-use, all-in-one injection system and can be used without a provider, making it a viable option for women in sub-Saharan Africa to self-inject.^4 5^

Self-injection has several important features for sub-Saharan women seeking to use contraception. First, there is an emphasis in the family planning field towards expanding access to self-care methods of contraception, that is, methods that women can use on their own or with minimal assistance from the healthcare system.^6^ The self-care approach is further embedded within the broader goal of contraceptive empowerment, in which a woman has the ability to decide for herself what she wants in relation to contraception and then acts on these preferences.^4 7^ Findings from qualitative studies investigating the preference for or acceptability of self-injection found that users appreciated the time and money savings, being able to bring units home for future use and not having to worry about stockouts, and increased privacy and confidentiality.^8 9^ Expansion of self-injection is also poised for broader health system benefits, including alleviation of time and resource burdens on healthcare providers, including task-shifting family planning services to community-level providers to expand access.^4 10 11^ With the onset of the COVID-19 pandemic, advocacy efforts surrounding self-care methods of contraception, including DMPA-SC, have intensified.^12^

Many reproductive health advocates and policymakers believe that self-injection represents the future of contraception within sub-Saharan Africa, and this promise appears to be reinforced by initial evidence showing increased prevalence of DMPA-SC in several sub-Saharan African settings.^13^ This promise has led to substantial donor funding and enthusiasm to roll-out DMPA-SC as a method of self-care,^3 14^ as well as a promising contraceptive method regardless of its self-injection capability, recognising the benefits of a lower hormone dose, smaller needle, and all-in-one application. Furthermore, large donors, supply coalitions, and governments are heavily invested in successful implementation of DMPA-SC given its potential to ease overburdened healthcare systems given the COVID-19 pandemic.

Despite the prominent advantages of self-injection for individuals and health systems, little is known about women’s attitudes towards self-injection. Yet, this information is critical, because for uptake and continuation of self-injection to accelerate in sub-Saharan Africa and globally, there must be demand for it. In this analysis, we present the first nationally and regionally representative findings on women’s knowledge and attitudes towards self-injection in Burkina Faso, Democratic Republic of Congo (DRC), Kenya and Nigeria, contextualising the results and weighing whether women’s preferences in these settings are reflected in current advocacy efforts.

Our data for this analysis come from Performance Monitoring for Action (PMA), which monitors reproductive health behaviours across eight countries in sub-Saharan Africa through annual surveys.^15^ Since 2016, PMA has included questions on DMPA-SC to help governments and donors understand prevalence of and patterns in uptake. Given the increasing use of DMPA-SC in many settings,^13^ the most recent PMA survey (PMA Phase 2) added questions to better understand women’s preferences, including knowledge of and attitudes towards self-injection. Full details of PMA, the study and sampling design, questionnaires and data are available from www.pmadata.org and Zimmerman *et al*.^15^ Further, we present data collected in winter 2020–2021 from four countries: Burkina Faso, DRC, Kenya, and Nigeria. Data collection in the DRC and Nigeria was limited to the provinces of Kinshasa and Kongo Central and the states of Kano and Lagos, respectively, and data are presented separately for each setting.

## Self-injection is fairly unknown to women in these contexts

Awareness or knowledge of a contraceptive method is a necessary component of demand for contraception (ie, women must know about a contraceptive method before they want to use it, and in turn, use it). To capture demand, data sources like the Demographic and Health Surveys (DHS) ask women if they know about specific contraceptive methods prior to assessing their use; however, these sources often do not ask about knowledge of delivery options, particularly self-injection,^16^ which is a relatively new practice.^5^ Therefore, information about women’s awareness of DMPA-SC’s capability to be self-injected is limited.

Few studies in sub-Saharan Africa have examined the acceptability and feasibility of self-injection from the client’s perspective^17–20^ and even fewer have specifically looked at demand.^8 20 21^ Those that did only assessed client preference and future recommendation for self-injection after clients had been trained and tried self-injecting as a participant in the study. To fill this gap and to better understand the potential demand for self-injection, the most recent PMA survey asked women, ‘Have you ever heard of injectables?’; if affirmative, women were additionally asked, ‘Have you heard that there is a type of injectable that you can inject yourself?’.

**Figure 1:**
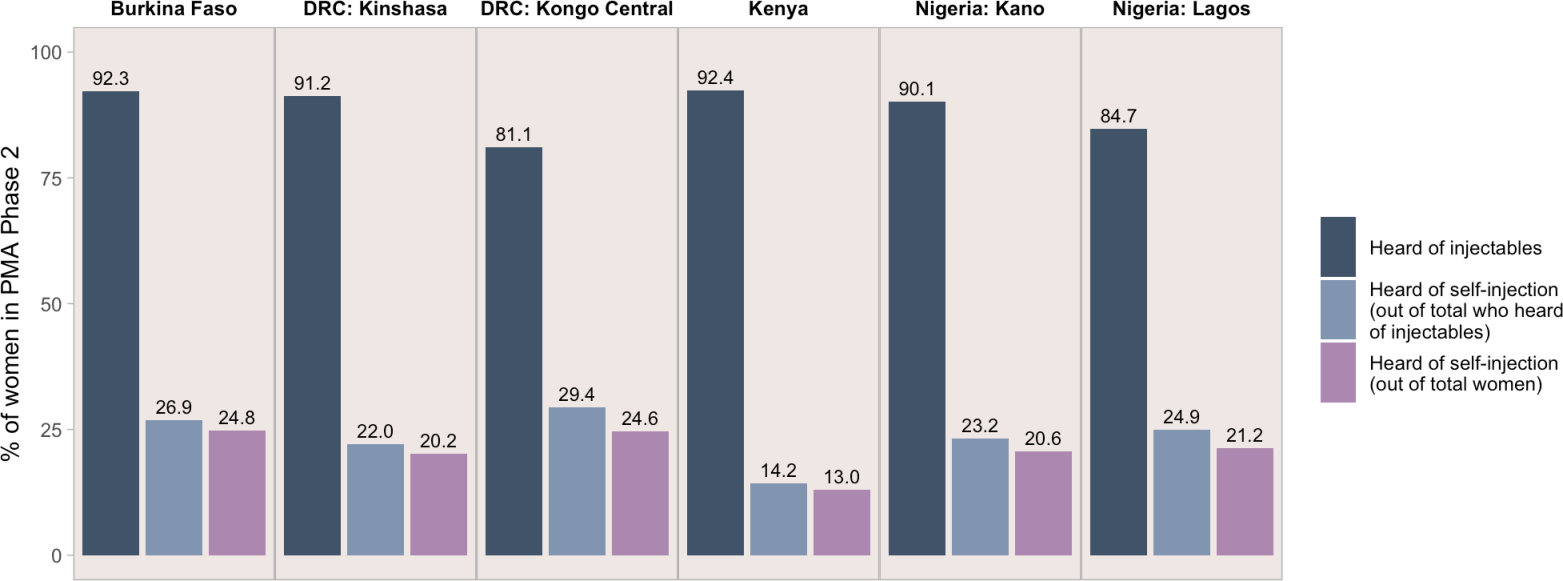
Percentage of women in PMA Phase 2 who had heard of injectables and self-injectables, by setting. PMA, Performance Monitoring for Action.

We present PMA Phase 2 data in figure 1 on knowledge of injectables and of self-injection. Our results show that self-injection is not a well-known mode of delivery. Over 80% of women had heard of injectables across all settings, ranging from 81.1% in DRC-Kongo Central to 92.4% in Kenya, yet only about one-fifth to one-fourth of women knew of the option to self-inject (ranging from 13.0% in Kenya to 24.8% in Burkina Faso) figure 1. When focusing only on women who had heard of injectables, knowledge of self-injection slightly increased; however, it remained consistently below 30%, and the difference between settings widened (ranging from 14.2% in Kenya to 29.3% in DRC-Kongo Central out of only those who had heard of injectables compared with 13.0% in Kenya to 24.8% in Burkina Faso out of all women).

The variation in the levels of awareness may be due to the different implementation strategies that countries adopted to integrate DMPA-SC into their family planning programmes. In Burkina Faso, one of countries that pioneered testing of DMPA-SC in sub-Saharan Africa, DMPA-SC is delivered alongside DMPA-IM at all levels of the health system.^3^ The DRC adopted a similar approach to offer expanded contraceptive choice to all family planning clients, advocating for DMPA-SC provision at the facility and community levels by clinical and non-clinical providers and training for self-injection with the support of medical and nursing students.^11 22^ Nigeria tackled both private and public sector delivery, using innovative supply-side solutions such as targeting high-volume contraceptive service facilities and in-facility trainings for providers.^18 23^ The country’s national guidelines for self-injection involve several visits to the provider to achieve proficiency before a user is given doses to self-administer without health provider supervision.^24^ In comparison, the roll-out of DMPA-SC in Kenya has been delayed and only recently expanded to public health facilities country-wide; self-injection has not yet been approved within this context.^25^ Given that self-injection is only available for those enrolled in research projects in Kenya,^25^ approximately 14% is actually relatively high awareness.

These different roll-out strategies were accompanied by varying approaches to demand generation for provider-administered DMPA-SC. In Burkina Faso, efforts focused on mass media campaigns and community-based communication activities, with a more targeted information, education, and communications approach to encourage the use of DMPA-SC among younger and rural women.^26^ The DRC leveraged youth campaigns and community-based distribution programmes, training CHWs to provide information and counsel on DMPA-SC via home visits and community outreach events.^11^ In Nigeria, demand-focused activities leveraged online and social media platforms in addition to traditional media approaches to promote general reproductive health services due to the restrictions on direct-to-consumer marketing of prescription medicines that prevented them from promoting DMPA-SC specifically.^18^

Expanding on these efforts, current investments in demand generation for self-injection are focused on dispelling myths about DMPA-SC and translating awareness to intention to use contraception. Notably, demand generation efforts for self-injection have only been implemented in one of the study countries—Nigeria. Two projects in Nigeria, the *Resilient & Accelerated Scale-up of DMPA-SC/Self-injection in Nigeria* in 2018^27^ and *Delivering Innovation in Self-Care* in 2020,^28^ aim to deliver DMPA-SC self-injection information using various channels such as interpersonal communication, social media, and community outreach and by engaging key actors, such as peers and their testimonies, partners, and social media influencers. Future activities will focus more on support mechanisms such as interactive counselling tools and follow-up to encourage uptake.

More research is required to better understand the levels of self-injection awareness observed across settings—monitoring trends over time can show whether demand is growing for this delivery modality. Furthermore, while increasing knowledge and awareness is a necessary step in demand generation, awareness is often not sufficient, as decisions to both use a contraceptive method at all and to use a specific method of contraception are influenced by an array of personal, interpersonal, and social factors.

## Progress in DMPA-SC use has been inconsistent over time and across countries, and few women have self-injected

**Figure 2:**
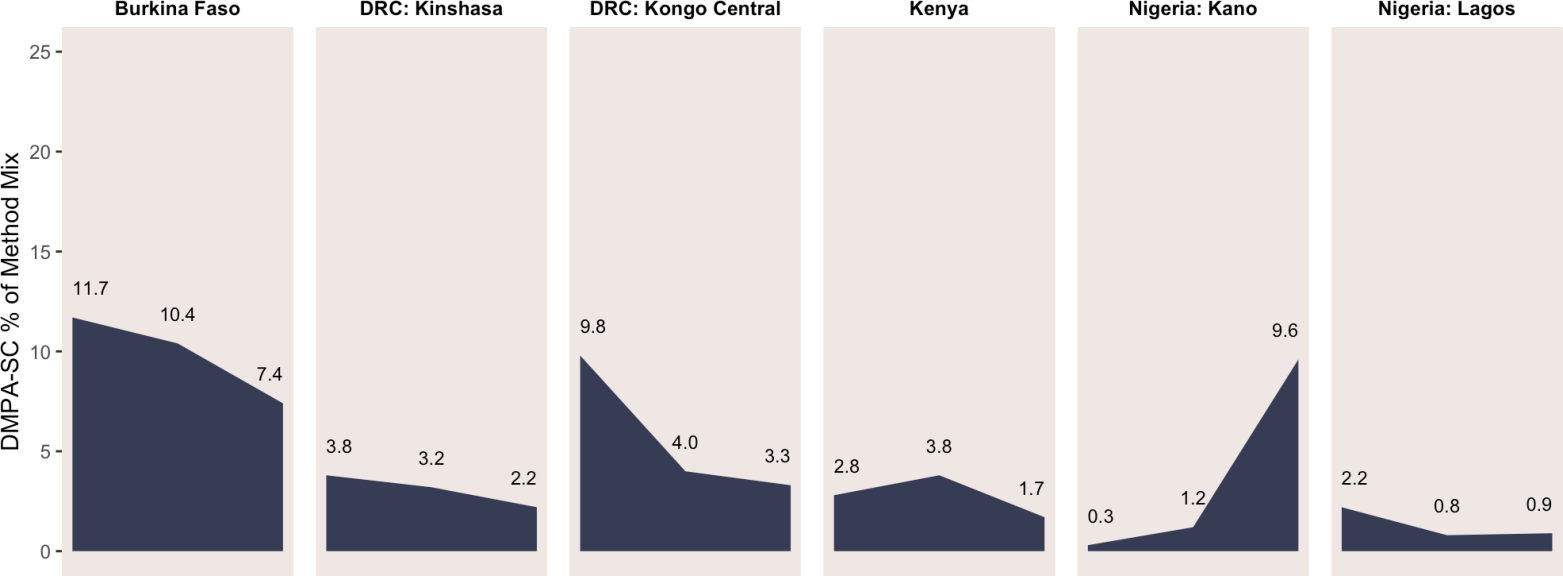
Trends in DMPA-SC as percentage of method mix over previous three PMA survey rounds, by setting, 2018–2021. We included the last three survey rounds for each setting: annual surveys from 2019 to 2021 in Burkina Faso and from 2018 to 2020 in Kenya and 2018, 2020 and 2021 surveys in DRC (Kinshasa and Kongo Central) and Nigeria (Kano and Lagos). DMPA-SC, subcutaneous depot medroxyprogesterone acetate; PMA, Performance Monitoring for Action.

Previous studies have reported increases in DMPA-SC use over time.^13 27^ To understand more recent trends in DMPA-SC use, we first examined the proportion of DMPA-SC use among total contraceptive users from 2018 to 2021 across settings (figure 2). DMPA-SC prevalence was assessed via a single item: ‘Was the injection administered via syringe or small needle?’; ‘small needle’ responses were then classified as DMPA-SC within the contraceptive method mix, whereas ‘syringe’ was classified as DMPA-IM.

We found that in Burkina Faso and DRC-Kongo Central, around 10% of contraceptive users reported DMPA-SC use at the beginning of this period (11.7% and 9.8%, respectively). In both contexts, however, the DMPA-SC proportion of the method mix dropped substantially over the three rounds of annual data collection to 7.4% in Burkina Faso and 3.3% in DRC-Kongo Central. In other settings, such as DRC-Kinshasa, the decline was more gradual, with the proportion of DMPA-SC users decreasing from 3.8% to 2.2% in the most recent round. Conversely, in Kenya and Nigeria-Lagos, observed trends in DMPA-SC were not linear. In Kenya, escalated proportions of use were observed in the middle data collection round (3.8%), whereas in Nigeria-Lagos, proportions dipped in the middle round (0.8%). The only setting reporting increased DMPA-SC use was Nigeria-Kano, where the proportion of DMPA-SC rose from 0.3% to 9.6% across three rounds.

Previous research using national PMA data showed increases in DMPA-SC use in Burkina Faso and the DRC between 2016 and 2019.^13^ Importantly, many DMPA-SC users were first-time users of modern contraception, ranging from 34.6% in the DRC to 54.7% in Burkina Faso,^13 29^ and switching to DMPA-SC from other contraceptive methods was less common.^29^ The stagnation in prevalence of DMPA-SC as a proportion of the method mix across settings, with the exception of Nigeria-Kano, may correspond with a similar slowing in modern contraceptive use levels; all settings except for Kongo Central displayed modest (<3%) rises in modern contraceptive prevalence across the three preceding PMA survey rounds.^30^ For DMPA-SC use to continue to rise, DMPA-SC should be offered to both non-users of contraception and users of other contraceptive methods, to ensure that women have access to a wide range of methods to use in line with their preferences. While supply constraints may partially explain stagnation, PMA data examining DMPA-SC provision at public and private facilities indicate increasing or stable stock between 2018 and 2020 (with exceptions of private facilities in Burkina Faso and Nigeria-Kano)^31^; however, decreases in DMPA-SC given the COVID-19 pandemic and global supply chain delays are certainly plausible, and the pandemic’s full impact on contraceptive commodities and usage has not yet been examined.

**Figure 3:**
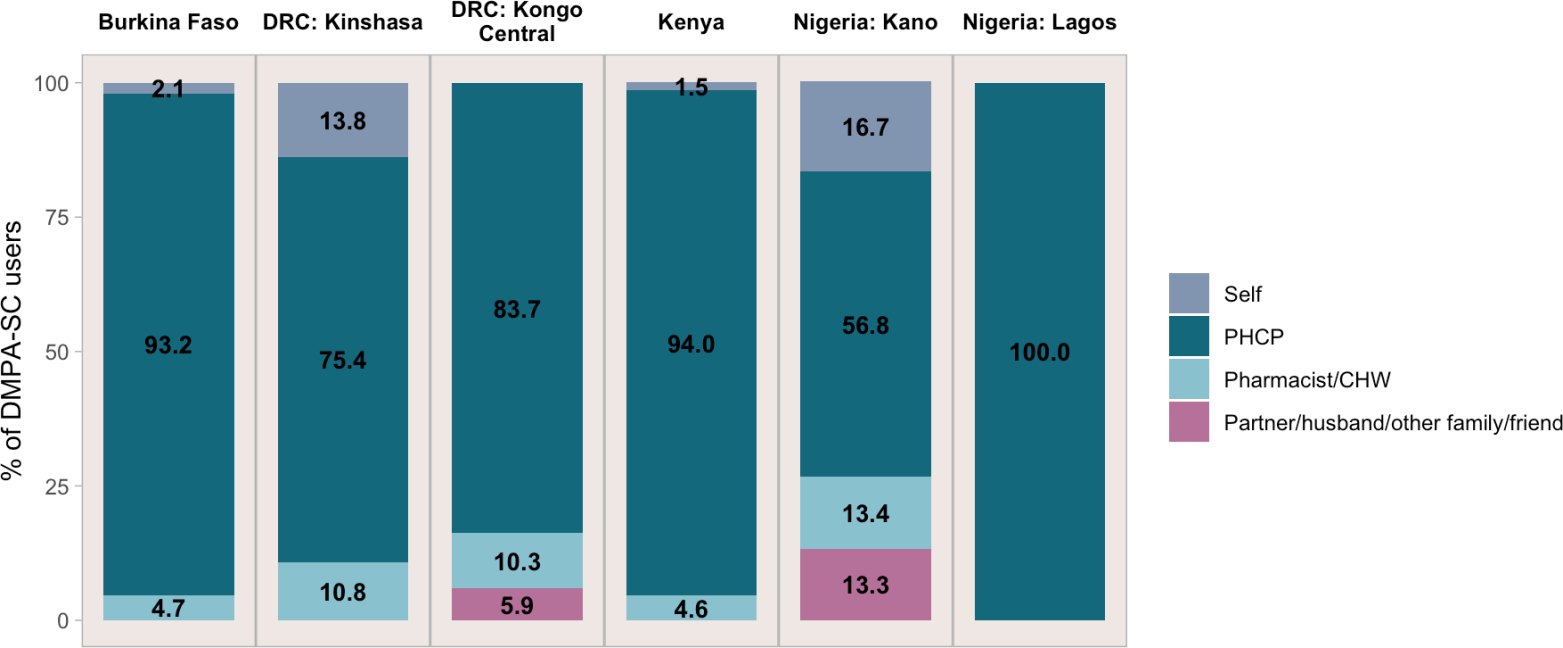
Person who administered the injection, among DMPA-SC users, by site. DMPA-SC, subcutaneous depot medroxyprogesterone acetate.

A major benefit of DMPA-SC surrounds its ability to be self-injected^4^; however, until now, national or regional level survey data surrounding self-injection has been non-existent. Using the first national and regional data surrounding self-injection, we asked all DMPA-SC users ‘Who administered the injection?’ with response categories including ‘self’; ‘partner/husband’; ‘other family/friend’; ‘doctor/nurse/midwife’; ‘pharmacist/drug shop employee’; ‘community health worker’; and ‘another user I know’. We found that among DMPA-SC users, the majority were injected by a professional healthcare provider (inclusive of doctor/nurse/midwife; figure 3). This proportion ranged from 56.8% in Nigeria-Kano to 100.0% in Nigeria-Lagos. Across all sites, a minority of respondents used self-injection. This proportion was highest in the Nigeria-Kano (16.7%) and DRC-Kinshasa (13.8%), whereas it was low in Burkina Faso (2.1%) and Kenya (1.5%). No users in the DRC-Kongo Central or Nigeria-Lagos reported self-injection.

Notably, our findings do not support suggestions that the majority of women using DMPA-SC wish to use it for its self-injection capability.^32^ The high proportions of self-injection in DRC-Kinshasa specifically may be explained by numerous campaigns to train women on self-injection in this setting.^33^ While these results are discouraging for self-injection advocates, they may point to a need for demand generation to raise awareness of the benefits of self-injection, as well as further training on self-injection for both women and providers so that women feel comfortable injecting and disposing of their method on their own.

## Most women prefer injectable contraceptives to be provider administered

To date, family planning programmes and advocacy efforts have largely assumed that one of the most appealing attributes of DMPA-SC is its potential for self-injection.^5^ To better understand demand specific to *self-injection*, rather than just for DMPA-SC, the latest PMA phase asked women who were not currently using contraception but wished to do so in the future, ‘What method do you think you will use?’. The injectable is one of the most popular contraceptive methods across sub-Saharan Africa^34^ so it is no surprise that 17.3%–44.8% of current non-users intending to use future contraception stated that they wanted to use the injectable (data not shown).

**Figure 4:**
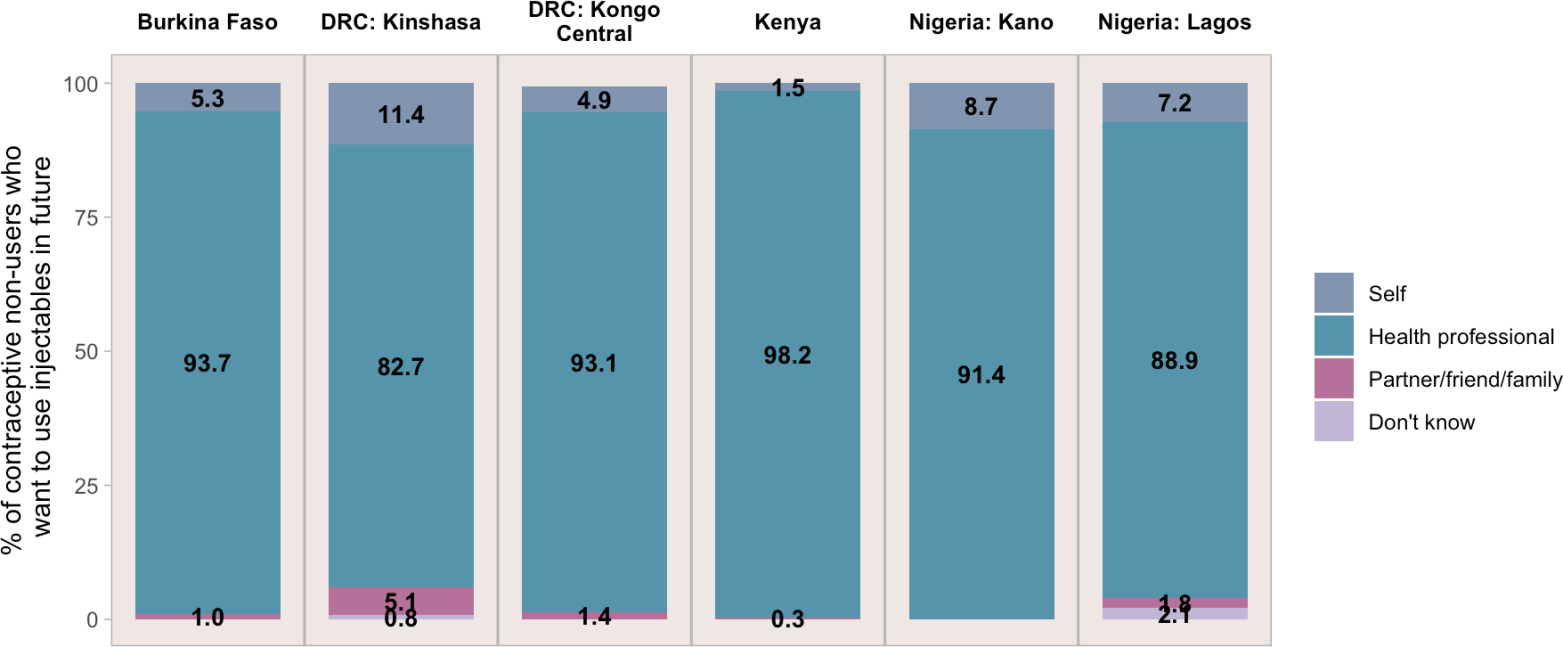
Person preferred to administer the injection, among contraceptive non-users saying they would want to use injectable in the future, by site. DRC, Democratic Republic of Congo.

We then asked women who thought they would use the injectable, ‘Who would you prefer to have administer the injectable?’ with response categories including ‘health professional’; ‘self’; ‘partner/friend/family’; and ‘don’t know’. Only 1.5%–11.4% of women intending to use the injectable stated that they would prefer to administer the injection themselves (figure 4). Instead, health professionals remained the most popular choice for injectable administration, ranging from 82.7% in DRC-Kinshasa to 98.2% in Kenya. A very small proportion of women in each setting stated that they would like a partner/friend/or family member to inject them (<2% except DRC-Kinshasa, where 5.1% stated this preference).

These results may speak to fear of the injectable modality as a self-care method. Though the injectable (both subcutaneous and intramuscular) remains a popular contraceptive method, women may not feel comfortable injecting themselves due to fear of needles, fear of making a mistake and not feeling competent enough to self-inject.^8 35^ Instead, they largely rely on the expertise of the trained healthcare provider. Similar hesitancies towards self-injection and preference for administration by a trained health provider have been noted with self-care injectable methods for long-acting antiretroviral therapy.^36^

Given the current knowledge base, it remains unclear whether increased training on self-injection will increase comfort and ease women’s qualms. In some cases, such as in Nigeria where two training sessions are required before a woman can take home DMPA-SC for self-injection, long learning periods may pose as a burden.^24^ Further research should seek to understand reasons for self-injection hesitancy. Moreover, these results speak to the reliance and credibility of healthcare providers as family planning experts within these study settings.

Another possibility is that women prefer the discrete nature of the injectable and do not feel that they are able to use the injectable covertly when self-injecting. It is estimated that 2%–15% of contraceptive users in sub-Saharan Africa use their method covertly (as assessed via the direct DHS question).^37^ Pending health concerns, side effects, and personal preferences, the injectable remains the most inconspicuous method to select if a woman wishes to use covertly, and self-injection could pose substantial risk that a woman’s partner learns of her use.

To date, qualitative research on motivators and barriers to self-injection has been limited. In Malawi and Uganda, Burke and colleagues explored barriers to DMPA-SC use for adolescents and/or covert users of contraception, where the authors hypothesised that disposal of the product after self-injection may pose substantial hindrance to continued use and found that indeed disposal placed increased risk and stress on self-injectors, specifically for those attempting to use covertly.^38^ Therefore, not only storage and usage should be considered when attempting to use this method covertly, but also safe disposal ramifications.

Notably, while interviewers were prompted when asking this question to explain that women would be trained on how to self-inject prior to injection, it is possible that this additional information was not relayed equitably across participants. As such, results for this item would be interpreted with caution, and further information is needed to understand whether women understood the training aspects required prior to electing to self-inject.

## Conclusion

Across nationally and regionally representative surveys from Burkina Faso, DRC (Kinshasa and Kongo Central), Kenya and Nigeria (Lagos and Kano), less than a third of women have heard about an injectable contraceptive that can be self-administered. Despite increases in DMPA-SC usage in some settings between 2016 and 2019, its prevalence is beginning to stagnate and decline. Overall, few current DMPA-SC users are self-injecting and the overwhelming majority of contraceptive non-users who wish to use an injectable in the future prefer it to be provider administered.

Attention is needed to understand these stagnating trends. Demand generation surrounding self-injection and continued training may be important for increasing interest in self-injectable DMPA-SC; of these contexts, to date, only Nigeria has promotional campaigns surrounding self-injection.^27 28^ Furthermore, shortages and understocking of DMPA-SC may impact provider willingness to prescribe this method and meet women’s desires regarding self-injection.^31^ Nonetheless, given the substantial advocacy and funding resources promoting DMPA-SC as a self-injectable method of contraception, learning more about women’s attitudes towards self-injection and DMPA-SC is critical. Investments in both quantitative and qualitative research must be made to understand women’s preferences for selecting a contraceptive method and hesitancy towards self-injecting.

## Data Availability

Data are available upon request from ww.pmadata.org.

## References

[1] Inject Sayana Press, 2022. Available: https://www.injectsayanapress.org/ [Accessed 31 Jan 2022].

[2] Subcutaneous DMPA access collaborative. Access collaborative Quarterly report: DMPA-SC by the numbers

[3] Stout A, Wood S, Barigye G, et al. Expanding access to injectable contraception: results from pilot introduction of subcutaneous depot medroxyprogesterone acetate (DMPA-SC) in 4 African countries. Glob Health Sci Pract 2018;6:55–72. 10.9745/GHSP-D-17-00250 29602866 PMC5878078

[4] Brady M, Drake JK, Namagembe A, et al. Self-care provision of contraception: evidence and insights from contraceptive injectable self-administration. Best Pract Res Clin Obstet Gynaecol 2020;66:95–106. 10.1016/j.bpobgyn.2020.01.003 32199705

[5] Kennedy CE, Yeh PT, Gaffield ML, et al. Self-administration of injectable contraception: a systematic review and meta-analysis. BMJ Glob Health 2019;4:1–13. 10.1136/bmjgh-2018-001350 PMC6528768 31179026

[6] World Health Organization. WHO guideline on self-care interventions for health and well-being. Geneva, 2021. https://www.who.int/publications/i/item/9789240030909

[7] Karp C, Wood SN, Galadanci H, Hadiza G, et al. 'I am the master key that opens and locks': Presentation and application of a conceptual framework for women's and girls' empowerment in reproductive health. Soc Sci Med 2020;258:113086. 10.1016/j.socscimed.2020.113086 32521413 PMC7369639

[8] Burke HM, Packer C, Buluzi M, et al. Client and provider experiences with self-administration of subcutaneous depot medroxyprogesterone acetate (DMPA-SC) in Malawi. Contraception 2018;98:405–10. 10.1016/j.contraception.2018.02.011 29706227

[9] Cover J, Ba M, Lim J, et al. Evaluating the feasibility and acceptability of self-injection of subcutaneous depot medroxyprogesterone acetate (DMPA) in Senegal: a prospective cohort study. Contraception 2017;96:203–10. 10.1016/j.contraception.2017.06.010 28673645 PMC6381449

[10] Di Giorgio L, Mvundura M, Tumusiime J, et al. Is contraceptive self-injection cost-effective compared to contraceptive injections from facility-based health workers? Evidence from Uganda. Contraception 2018;98:396–404. 10.1016/j.contraception.2018.07.137 30098940 PMC6197841

[11] Hernandez JH, Akilimali P, Glover A, et al. Task-shifting the provision of DMPA-SC in the Dr Congo: perspectives from two different groups of providers. Contraception 2018;98:449–53. 10.1016/j.contraception.2018.07.002 30031000 PMC6197837

[12] Nanda K, Lebetkin E, Steiner MJ, et al. Contraception in the era of COVID-19. Glob Health Sci Pract 2020;8:166–8. 10.9745/GHSP-D-20-00119 32312738 PMC7326510

[13] Anglewicz P, Akilimali P, Guiella G, et al. Trends in subcutaneous depot medroxyprogesterone acetate (DMPA-SC) use in Burkina Faso, the Democratic Republic of Congo and Uganda. Contracept X 2019;1:100013. 10.1016/j.conx.2019.100013 32550528 PMC7286145

[14] PATH. PATH welcomes $10.5 million grant to expand contraceptive choice and access, 2017. Path media cent. Available: https://www.path.org/media-center/path-welcomes-105-million-grant-to-expand-contraceptive-choice-and-access/ [Accessed 07 Feb 2022].

[15] Zimmerman L, Olson H, et al, PMA2020 Principal Investigators Group. PMA2020: rapid Turn-Around survey data to monitor family planning service and practice in ten countries. Stud Fam Plann 2017;48:293–303. 10.1111/sifp.12031 28885679 PMC6084342

[16] DHS. Demographic and Health Surveys: Model Woman’s Questionnaire, 2016: 1–75.

[17] Sherpa LY, Tinkari BS, Gentle P, et al. A prospective cohort study to assess the acceptability of Sayana press among 18-49-year-old women in Nepal. Contraception 2021;104:623–7. 10.1016/j.contraception.2021.07.009 34280441

[18] Liu J, Schatzkin E, Omoluabi E, et al. Introducing the subcutaneous depot medroxyprogesterone acetate injectable contraceptive via social marketing: lessons learned from Nigeria's private sector. Contraception 2018;98:438–48. 10.1016/j.contraception.2018.07.005 30071196 PMC6197840

[19] Polis CB, Nakigozi GF, Nakawooya H, et al. Preference for Sayana® press versus intramuscular Depo-Provera among HIV-positive women in Rakai, Uganda: a randomized crossover trial. Contraception 2014;89:385–95. 10.1016/j.contraception.2013.11.008 24332432

[20] Burke HM, Chen M, Buluzi M, et al. Factors affecting continued use of subcutaneous depot medroxyprogesterone acetate (DMPA-SC): a secondary analysis of a 1-year randomized trial in Malawi. Glob Health Sci Pract 2019;7:54–65. 10.9745/GHSP-D-18-00433 30894394 PMC6538126

[21] Khan S, Grady B, Tifft S. Estimating demand for a new contraceptive method: projections for the introduction of Sayana press. Int J Gynaecol Obstet 2015;130 Suppl 3:E21–4. 10.1016/j.ijgo.2015.03.020 26092777

[22] PATH. Self-injection of DMPA-SC in Ghana, Malawi, DRC, Senegal, and Uganda: increasing access, improving continuation, and empowering women, 2019. Learn action Netw webinars. Available: https://path.ent.box.com/s/2kuhosn119m469hr7jntvzdsueykl0m9/file/403115068792

[23] Liu J, Shen J, Schatzkin E, et al. Accessing DMPA-SC through the public and private sectors in Nigeria: users’ characteristics and their experiences. Gates Open Res 2019;2:73. 10.12688/gatesopenres.12890.2

[24] Health NFM of. National guidelines for the introduction and scale-up of DMPA-SC self-injection, 2019. Available: https://health.gov.ng/doc/National_Guidelines_For_The_Introduction_And_Scale-Up_Of_DMPA-SC_Self_Injection.pdf

[25] PATH JSI. DMPA-SC Access Collaborative Country Brief: Kenya’s journey to DMPA-SC and self-injection scale-up, 2021. Available: https://fpoptions.org/wp-content/uploads/Kenya-DMPA-SC-country-brief-PATH-JSI-2021.pdf

[26] PATH. Subcutaneous DMPA-Introduction: practical guidance from path, 2018.

[27] Osinowo K, Sambo-Donga F, Ojomo O, et al. Resilient and accelerated scale-up of subcutaneously administered Depot-Medroxyprogesterone acetate in Nigeria (RASuDiN): a Mid-line study in COVID-19 era. Open Access J Contracept 2021;12:187–99. 10.2147/OAJC.S326106 34880691 PMC8648267

[28] PSI. Delivering innovation in self-care (disc). Available: https://www.psi.org/project/disc/ [Accessed 07 Feb 2022].

[29] Anglewicz P, Larson E, Akilimali P, et al. Characteristics associated with use of subcutaneous depot medroxyprogesterone acetate (DMPA-SC) in Burkina Faso, Democratic Republic of Congo, and Uganda. Contracept X 2021;3:100055. 10.1016/j.conx.2021.100055 33554107 PMC7846921

[30] PMA Datalab. Performance monitoring for action, 2020.

[31] Magalona S, Wood SN, Makumbi FM, et al. DMPA-SC stock: Cross-site trends by facility type. Contraception X 2022;4. 10.1016/j.conx.2022.100075 PMC9046645 35493973

[32] Nai D, Tobey E, Fuseini K, et al. What distinguishes women who choose to Self-Inject? A prospective cohort study of subcutaneous depot medroxyprogesterone acetate users in Ghana. Glob Health Sci Pract 2022;10:1–12. 10.9745/GHSP-D-21-00534 PMC8885352 35294390

[33] Bertrand JT, Bidashimwa D, Makani PB, et al. An observational study to test the acceptability and feasibility of using medical and nursing students to instruct clients in DMPA-SC self-injection at the community level in Kinshasa. Contraception 2018;98:411–7. 10.1016/j.contraception.2018.08.002 30120925 PMC6197832

[34] Ahmed S, Choi Y, Rimon JG, et al. Trends in contraceptive prevalence rates in sub-Saharan Africa since the 2012 London Summit on family planning: results from repeated cross-sectional surveys. Lancet Glob Health 2019;7:e904–11. 10.1016/S2214-109X(19)30200-1 31109881 PMC6560024

[35] Cover J, Ba M, Drake JK, et al. Continuation of self-injected versus provider-administered contraception in Senegal: a nonrandomized, prospective cohort study. Contraception 2019;99:137–41. 10.1016/j.contraception.2018.11.001 30439358 PMC6367564

[36] Simoni JM, Beima-Sofie K, Wanje G, et al. “Lighten This Burden of Ours”: Acceptability and preferences regarding injectable antiretroviral treatment among adults and youth living with HIV in Coastal Kenya. J Int Assoc Provid AIDS Care 2021;20:232595822110005–10. 10.1177/23259582211000517 PMC7952847 33685272

[37] Choiriyyah I, Becker S. Measuring women's covert use of modern contraception in cross-sectional surveys. Stud Fam Plann 2018;49:143–57. 10.1111/sifp.12053 29845621

[38] Burke HM, Packer C, Wando L, et al. Adolescent and covert family planning users’ experiences self-injecting contraception in Uganda and Malawi: implications for waste disposal of subcutaneous depot medroxyprogesterone acetate. Reprod Health 2020;17:1–13. 10.1186/s12978-020-00964-1 32746860 PMC7396890

